# The influence of chorioamnionitis on respiratory drive and spontaneous breathing of premature infants at birth: a narrative review

**DOI:** 10.1007/s00431-024-05508-4

**Published:** 2024-04-01

**Authors:** Timothy J. R. Panneflek, Kristel L. A. M. Kuypers, Graeme R. Polglase, Douglas P. Derleth, Janneke Dekker, Stuart B. Hooper, Thomas van den Akker, Arjan B.te Pas

**Affiliations:** 1 Division of Neonatology, Department of Paediatrics, Willem-Alexander Children’s Hospital, Leiden University Medical Centre, P.O. Box 9600, 2300 RC Leiden, Netherlands; 2 The Ritchie Centre, Hudson Institute of Medical Research, Clayton, VIC Australia; 3 Department of Obstetrics and Gynaecology, Monash University, Clayton, VIC Australia; 4 Department of Paediatric and Adolescent Medicine, Mayo Clinic, Rochester, MN USA; 5 Department of Obstetrics, Leiden University Medical Centre, Leiden, The Netherlands

**Keywords:** Chorioamnionitis, Breathing, Resuscitation, Neonatology, Inflammation

## Abstract

Most very premature infants breathe at birth but require respiratory support in order to stimulate and support their breathing. A significant proportion of premature infants are affected by chorioamnionitis, defined as an umbrella term for antenatal inflammation of the foetal membranes and umbilical vessels. Chorioamnionitis produces inflammatory mediators that potentially depress the respiratory drive generated in the brainstem. Such respiratory depression could maintain itself by delaying lung aeration, hampering respiratory support at birth and putting infants at risk of hypoxic injury. This inflammatory-mediated respiratory depression may contribute to an association between chorioamnionitis and increased requirement of neonatal resuscitation in premature infants at birth. This narrative review summarises mechanisms on how respiratory drive and spontaneous breathing could be influenced by chorioamnionitis and provides possible interventions to stimulate spontaneous breathing.

*Conclusion*: Chorioamnionitis could possibly depress respiratory drive and spontaneous breathing in premature infants at birth. Interventions to stimulate spontaneous breathing could therefore be valuable.

**Table:** 

**What is Known:** *• A large proportion of premature infants are affected by chorioamnionitis, antenatal inflammation of the foetal membranes and umbilical vessels.*	
**What is New:** *• Premature infants affected by chorioamnionitis might be exposed to higher concentrations of respiratory drive inhibitors which could depress breathing at birth.* *• Premature infants affected by chorioamnionitis seem to be associated with a higher and more extensive requirement of resuscitation at birth. *

**Supplementary Information:**

The online version contains supplementary material available at 10.1007/s00431-024-05508-4.

## Introduction

Premature infants have an immature respiratory system at birth and often require respiratory support to aerate their lungs and establish effective continuous breathing [1]. However, the ability of non-invasive respiratory support to assist lung aeration is largely dependent on the level of spontaneous breathing activity [2]. Clinical and experimental studies have shown that the larynx is mainly closed during apnoea and only opens during a spontaneous breath [3, 4]. As closure of the larynx prevents air from entering the lungs, it hampers the ability of non-invasive respiratory support to assist with lung aeration and oxygenation of the infant. Adequate spontaneous breathing is therefore pivotal for effective non-invasive ventilation.

While there are several factors that may promote spontaneous breathing (tactile stimulation and caffeine), premature infants also face factors that depress breathing, such as hypoxia and inflammation [1]. A large proportion of premature infants are antenatally exposed to inflammation of the foetal membranes and umbilical vessels, which fall under the umbrella term ‘chorioamnionitis’ [5]. Since chorioamnionitis-induced inflammation appears to depress breathing both before and after birth, premature infants affected by chorioamnionitis likely face the combined problems of an immature respiratory system and inflammatory-mediated respiratory depression, which synergistically could suppress effective continuous breathing of neonates at birth [6–9].

In this narrative review, we discuss (i) mechanisms of how chorioamnionitis might affect respiratory drive and spontaneous breathing in premature infants and (ii) possible interventions.

## Methods

We performed a literature search on MEDLINE for (pre)clinical studies investigating the effects and mechanisms of chorioamnionitis and inflammatory/hypoxic mediators on respiratory drive and spontaneous breathing parameters in newborns (Supplementary Fig. 1). A cross-check was made using the reference list of included articles and relevant reviews to identify any studies not included in the primary search. Additional literature searches were performed on MEDLINE for the effect of the possible interventions on chorioamnionitis and breathing parameters (Supplementary Fig. 2). All studies that reported data on chorioamnionitis and spontaneous breathing parameters (in premature infants) were included and reported in a narrative summary without grading or pooling of the effect sizes.

## Results and discussion

### Defining and identifying chorioamnionitis

Prior to birth, chorioamnionitis can be identified clinically via the ‘suspected Triple I criteria’,^1^ which are aimed to detect *i*ntra-uterine/amniotic *i*nflammation, *i*nfection, or both (Triple I) [10]. Of the Triple I, only intra-amniotic inflammation, an immunologic reaction to micro-organisms or stressful situations (such as hypoxia), is associated with adverse outcomes, consisting of a composite of stillbirth, neonatal death, respiratory distress syndrome, intraventricular haemorrhage, necrotising enterocolitis and sepsis [11, 12].

After birth, the diagnosis of chorioamnionitis is either determined or confirmed by placental histology with evidence of antenatal inflammation affecting the infant (i.e. histological chorioamnionitis; the gold standard) [10, 13]. Although the suspected Triple I criteria could identify all intra-amniotic infection cases (specificity 100%), the criteria are not particularly sensitive (36–71%) or specific (41–71%) in diagnosing histological chorioamnionitis [14, 15].

This indicates that, while clinical chorioamnionitis is associated with a maternal systemic inflammatory response, the suspected Triple I criteria will lead to under-diagnosis of histological chorioamnionitis cases. In these subclinical cases, the foetus might still be exposed to antenatal inflammation, which potentially affects their breathing at birth, in the absence of a systemic maternal inflammatory response.

### Chorioamnionitis and breathing effort at birth

Observational studies report that premature infants affected by any type of chorioamnionitis require resuscitation more often and more extensively at birth (Table 1) [9, 16–27]. The increased requirement of resuscitation consisted of associations between chorioamnionitis and any resuscitation/ventilatory support in four studies [17, 20, 21, 23]; oxygen supplementation in two studies [9, 23]; and intubation, cardiopulmonary resuscitation or volume expansion in six studies [16, 18, 19, 21, 24, 26, 27]. Pietrasanta et al. observed that only a foetal inflammatory response (i.e. funisitis) was associated with a higher requirement of need for resuscitation/oxygen supplementation at birth after adjusting for gestational age and that sole inflammation of the foetal membranes was not associated with these outcomes [23]. In our retrospective case–control study, there was no significant difference between premature infants affected by chorioamnionitis and matched controls with regard to the incidence of delivery room intubation [9]. Similarly, Gisslen et al., Pappas et al. and Sarkar et al. also reported no significant association between chorioamnionitis and the need for intubation, chest compressions and/or epinephrine [20, 22, 25]. Although findings are conflicting, the majority of available data indicate that chorioamnionitis is associated with an additional requirement of resuscitation at birth.

**Table 1:** Summary of studies reporting on chorioamnionitis and resuscitation at birth

Author & Year	Country	Study design	Time	Population	Sample size (infants)	Chorioamnionitis type	Resuscitation at birth	Unadjusted odds ratio (95%-CI)	Gestational age adjusted odds ratio (95%-CI) or adjusted p-value
Botet 2010	Spain	Matched controls	Prospective	Premature infants ≤ 1500 g	328	Clinical	Endotracheal intubation		2.02 (1.30–3.14)^1^
Ecevit 2014	Turkey	Cohort	Retrospective	Premature infants with premature rupture of membranes < 37 weeks’ gestation	58	Histological	Need of resuscitation	15.00 (2.64–84.79)^*^	
Figueras-Aloy 2001	Spain	Cohort	Cross-sectional	Mechanically ventilated premature infants between 25–26 weeks’ gestation	54	Clinical	Endotracheal tube resuscitation	6.86 (1.84–25.61)^*^	
García-Muñoz Rodrigo 2014	Spain	Cohort	Prospective	Premature infants < 32 weeks’ gestation and ≤ 1500g	8330	Clinical	Endotracheal intubation, chest compressions and/or the administration of medications	2.01 (1.80–2.55)^*^	
Gisslen 2016	USA	Observational	Prospective	Premature infants between 32–36 weeks’ gestation	477	Histological	Oxygen supplementation^1^ CPAP^2^ PPV^3^ Any ventilation support^4^ Intubation^5^	0.63 (0.34–1.14)^1^ 2.62 (1.51–5.54)^2*^ 1.65 (0.87–3.13)^3^ 2.04 (1.23–3.39)^4*^ 3.29 (0.20–53.05)^5^	
González-Luis 2002	Spain	Matched cohort	Retrospective	Premature infants < 1500 g	135	Clinical or subclinical	Any resuscitation^1^ Bag and mask ventilation^2^ Intubation^3^		4.18 (1.85–9.46)^1*^ 2.69 (0.68–10.55)^2^ 2.86 (1.36–6.51)^3*^
Panneflek 2023	The Netherlands	Matched cohort	Retrospective	Premature infants < 30 weeks’ gestation	92	Clinical	Intubation^1^ Average oxygen supplementation (%)^2^		3.33 (0.92–12.11)^1^ *p* = 0.036^2*^
Pappas 2015	USA	Cohort	Retrospective	Premature infants < 27 weeks’ gestation	2390	Histological and clinical	Intubation, chest compressions and/or epinephrine	0.95 (0.80–1.14)	
Pietrasanta 2019	Italy	Cohort	Prospective	Premature infants < 35 weeks’ gestation ≤ 1500 g	807	Histological	Resuscitation (at least ventilation with mask)^1^ Oxygen supplementation^2^	2.67 (1.80–4.02)^*^ 2.03 (1.42–2.90)^*^	
Ryan 2019	Ireland	Cohort	Retrospective	Premature infants < 32 weeks’ gestation or < 1500 g	499	Histological	Bag-mask ventilation^1^ Intubation^2^ Surfactant administration^3^	1.86 (1.06–3.27)^1*^ 3.75 (2.46–5.70)^2*^ 3.68 (2.35–5.77)^3*^	*p* = 0.491^1^ *p* = 0.006^2*^ *p* = 0.023^3*^
Sarkar 2005	USA	Observational	Prospective	Premature infants < 28 weeks’ gestation	62	Histological	Cardiopulmonary and volume resuscitation	6.09 (0.28–132.26)	
Soraisham 2014	Canada	Cohort	Retrospective	Premature infants < 33 weeks’ gestation	8033	Clinical	Chest compressions ± epinephrine administration	1.47 (1.14–1.90)^*^	
Strunk 2019	Australia	Cohort	Retrospective	Premature infants < 30 weeks’ gestation	1089	Histological	Intubation	1.81 (1.38–2.37)^*^	

*Statistically significant difference between groups

### Potential mechanisms of action

Initially, chorioamnionitis-induced inflammation is thought to activate the immune system via pathogen-associated molecular patterns and damage-associated molecular patterns [12, 28]. Both pathways potentially increase the activity of secretory phospholipase A_2_ activity; release pro-inflammatory cytokines that could modulate respiratory drive (such as interleukin-1β and interleukin-6); and induce both prostaglandin-H-synthase-2 (consisting of cyclooxygenase and peroxidase moieties) and microsomal prostaglandin E synthase-1 [29–31]. Secretory phospholipase A_2_ liberates arachidonic acid from the glycerophospholipid of the membrane, which the induced enzymes collectively metabolise into prostaglandin E_2_ (PGE_2_) during inflammation [29, 30, 32]. Additionally, this inflammation also leads to lower partial pressure of oxygen in arterial blood [7]. Experimental and clinical data suggest that the inflammatory and hypoxic mediators, PGE_2_ and adenosine, inhibit the respiratory drive generated in the brainstem, which depresses both foetal and postnatal breathing [7, 33–37].

In a retrospective case–control study, we observed that premature infants affected by chorioamnionitis had reduced breathing effort and oxygenation when compared to matched controls with similar gestational age [9]. Our observations were consistent with the findings of a previous study that reported lower spontaneous breathing rates in very low birthweight infants affected by chorioamnionitis, though they did not adjust for gestational age [8]. Presumably, a lower rate of spontaneous breathing, caused by the release of inhibitory mediators such as PGE_2_ and adenosine, delays lung aeration and thereby the increase in oxygenation, since breathing is the most important driver of lung aeration [38]. Moreover, during respiratory depression, the larynx remains predominantly closed, thereby compromising the effectiveness of non-invasive respiratory support, leading to a further delay in lung aeration [2–4].

Theoretically, delayed lung aeration might also decrease the clearance of circulating and central PGE_2_ and adenosine. Lung aeration triggers an increase in pulmonary blood flow via widespread pulmonary vasodilatation [38]. As PGE_2_ and other bioactive prostaglandins are rapidly metabolised from the circulation as these molecules pass through the lung, pulmonary PGE_2_ clearance likely increases at birth due to increased pulmonary blood flow [38, 39]. Similarly, as adenosine is released during hypoxia, circulating and central adenosine concentrations likely share an inverse relationship with oxygenation (a reflection of the surface area available for gas exchange), which rises following lung aeration [1, 40].

Therefore, it is possible that respiratory depression at birth can turn into a self-sustaining inhibitory cycle, hampering the ability to provide respiratory support by means of a closed larynx during apnoea [3, 4]. This inhibitory cycle could continue until an infant gets so hypoxic that normal reflexes are lost, causing the laryngeal dilator muscles to relax and allowing the lungs to be aerated by non-invasive ventilation or mechanical ventilation following intubation [3]. Considering that respiratory support is mostly aimed at stimulating and supporting spontaneous breathing, current measures might be less effective in premature infants affected by chorioamnionitis, as the infants might be exposed to higher concentrations of respiratory drive inhibitors and could therefore be more depressed at birth, which requires more (extensive) resuscitation.

This resuscitation is potentially injurious to premature infants in general, as their lungs are prone to ventilation-induced lung injury, but especially in premature infants affected by chorioamnionitis that might have altered lung function [41, 42]. Experimental studies in lambs have shown that antenatal inflammation, via the release of mediators like PGE_2_, could increase surfactant production and improve lung compliance after birth, thereby inducing lung maturation [43]. However, the antenatal inflammation possibly maturing the lungs might also sensitise the premature lung to respond with a heightened inflammatory response to ventilation associated with lung injury [44, 45]. Despite possible lung maturation, chorioamnionitis is associated with lower tidal volume at birth, possibly indicating that respiratory drive might play a larger role at birth than mechanisms of lung function (maturation or injury) in premature infants affected by chorioamnionitis [9].

### Possible interventions

A possible way to improve respiratory drive and spontaneous breathing at birth is to decrease the concentration of PGE_2_. Although decreasing PGE_2_ concentrations might not be essential for establishing breathing at birth, it does promote continuous breathing and is inversely correlated with the degree of respiratory activity observed in the perinatal period [46, 47]. Indomethacin, a non-steroidal anti-inflammatory drug, inhibits PGE_2_ synthase, resulting in an increase of the depth and rate of foetal breathing movements [48]. In addition, there is an increase of depth of breathing at birth (increase in tidal volumes), which is possibly due to increased peak phrenic nerve activity [48–50]. However, indomethacin also inhibits surfactant production and increases the need for surfactant and additional ventilatory support in neonates [48, 50–53]. Thus, a safer alternative to indomethacin is warranted.

Paracetamol, an acetanilide derivative, also decreases central PGE_2_ concentrations [54, 55]. Antenatal paracetamol administration just prior to birth has the potential to improve breathing at birth and is safe to use during labour without any reported foetal or maternal adverse events in six randomised controlled trials [56–61]. Although the exact mechanism is unclear, the following mechanisms can be involved: (i) depleting glutathione, necessary for paracetamol metabolism, which limits the last step of PGE_2_ synthesis; (ii) scavenging peroxynitrite, which is necessary for cyclooxygenase activity; and (iii) inactivating cyclooxygenase activity, by acting as a reducing co-substrate for the peroxidase moiety [62, 63].

It has been demonstrated that antenatal paracetamol administered directly to the foetal lamb primarily increased the depth (by 221%) and secondarily rate (by 83%) of foetal breathing [64]. However, paracetamol administered to pregnant women, which crosses the placenta and results in similar concentrations between foetus and mother, did not significantly increase the rate of foetal breathing in premature foetuses [65, 66]. This discrepancy between experimental and clinical can be explained by the fact that in the clinical study, paracetamol concentrations were barely at therapeutic levels at the time of foetal activity assessment, whereas they were supraphysiologic in the experimental studies [64, 66, 67]. Also, less sensitive equipment was used to detect foetal breathing in the clinical setting, compared to the experimental setting [65, 66]. An observational study in premature infants exposed to antenatal paracetamol 24 h prior to birth showed less acute rescue interventions at birth, predominantly due to less need for surfactant administration [68]. In this study, infants exposed to antenatal paracetamol also required lower maximum ventilation pressures after birth compared to matched controls and antenatal paracetamol exposure correlated positively with all Apgar scores at birth [68]. While caution is needed when interpreting observational studies, there seems to be an association between improved respiratory function in premature infants and antenatal paracetamol exposure.

Next to paracetamol, antenatal N-acetylcysteine, which crosses the placenta and achieves predictable plasma concentrations in the foetus, could also improve spontaneous breathing [69]. N-Acetylcysteine is an antidote to paracetamol overdoses, because it replenishes glutathione used for paracetamol metabolism, but also inhibits prostaglandin synthesis and potentiates the effect of other PGE_2_ synthase inhibitors [70, 71]. In a randomised controlled trial (*n* = 67), antenatal N-acetylcysteine increased Apgar scores and decreased the requirement of invasive ventilation and surfactant administration, in premature infants affected by confirmed Triple I compared to placebo without any serious adverse events, but 15% of pregnant women reported minor adverse events, comprising of nausea and vomiting, transient chest tightness and a hot flushes [72]. Since all glutathione concentration parameters were similar between both groups in this study, PGE_2_ inhibition might have been partly responsible for the decreased requirement of resuscitation at birth after antenatal N-acetylcysteine administration [72].

Another way to reduce the respiratory depression associated with chorioamnionitis is to focus on reducing the inhibitory effects of hypoxia, because hypoxic mediation of antenatal inflammation both before and after birth may increase PGE_2_ and adenosine concentrations [7, 9, 36]. Hence, inhibiting or mitigating the biological action of PGE_2_, as described above, and adenosine may have therapeutic benefits. Indeed, decreasing adenosine concentrations is additive to establishing continuous breathing at birth [73]. Adenosine concentrations can be influenced by administration of caffeine, a nonselective adenosine receptor antagonist, and supplemental oxygen [74, 75]. These interventions counteract adenosine-dependent and adenosine-independent mechanisms involved in the hypoxic inhibition of breathing and improve breathing effort and oxygenation [74, 75]. While caffeine may be administered antenatally, its effect is partly dependent on maternal caffeine consumption during pregnancy; hence, postnatal caffeine administration might be more pragmatic [76].

Additional considerations for potential interventions could consist of anti-inflammatory agents known to suppress antenatal inflammation in experimental studies, such as glucocorticoids, cytokine suppressors, melatonin and magnesium sulphate, but data on their effect on respiratory drive and spontaneous breathing at birth is scarce [77–82]. These agents could lower PGE_2_ and adenosine levels by acting on precursors of their inflammatory cascade [77–82]. As PGE_2_ and adenosine are important messengers also involved in multiple organ systems, lowering normal responses to hypoxia and inflammation might have negative consequences on the brain or other organs (e.g. the cardiovascular system). Nevertheless, systematic reviews and meta-analysis demonstrated no neurodevelopmental complications of early postnatal paracetamol and caffeine administration [83, 84]. Therefore, lowering possibly elevated levels of PGE_2_ and adenosine after birth does not seem to affect brain development and could potentially assist in preventing potentially injurious ventilation.

Lastly, postponing cord clamping until lung aeration has been established (physiologically based cord clamping (PBCC)) could mitigate the reduced oxygenation experienced by premature infants at birth affected by chorioamnionitis. PBCC permits lung aeration during placental transfusion of oxygen-rich blood and differs from delayed cord clamping by clamping the cord after the infants have been stabilised and not after an arbitrary time point has passed, which accelerates neonatal stabilisation of premature infants at birth [85, 86]. PBCC in lambs affected by antenatal inflammation improved cardiovascular circulatory stability compared to immediate cord clamping by preventing a fall in systemic oxygen saturation [87]. However, continuing the influx of inflammatory mediators (e.g. PGE_2_ and adenosine), reaching the brainstem of the infant could overwhelm an infant’s clearance of these mediators and depress breathing [88]. Thus, future research on the effects of PBCC on spontaneous breathing of premature infants affected by chorioamnionitis would be beneficial.

## Conclusion

Chorioamnionitis possibly delays lung aeration via an inflammatory-mediated respiratory depression, which hampers non-invasive respiratory support and potentially reduces oxygenation in premature infants at birth. Clinicians should be aware that premature infants affected by chorioamnionitis might therefore require resuscitation more often and more extensively at birth. As premature infants already have difficulty with breathing spontaneously and effectively at birth, stimulating and supporting spontaneous breathing in these infants with optimal oxygenation management and possible pharmaceutical agents might reduce the need for invasive ventilation. The role of intact cord resuscitation in these premature infants is unclear, and more research is needed to determine whether continuing influx of inflammatory mediators influences respiratory drive at birth.

### Supplementary Information

Below is the link to the electronic supplementary material.

Supplementary file1 (DOCX 13 KB)

Supplementary file2 (DOCX 15 KB)

## Data Availability

No datasets were generated or analysed during the current study.

## Abbreviations

PGE_2_: Prostaglandin E_2_
PBCC: Physiologically based cord clamping

## Footnotes

1 These criteria consist of fever and one of the following symptoms: leukocytosis, purulent vaginal discharge or foetal tachycardia. 10.Peng CC, Chang JH, Lin HY, Cheng PJ, Su BH. Intrauterine inflammation, infection, or both (Triple I): a new concept for chorioamnionitis. Pediatr Neonatol. 2018;59(3):231–7.
