# Supplementary material for: The influence of chorioamnionitis on respiratory drive and spontaneous breathing of premature infants at birth: a narrative review
Source: Eur J Pediatr. 2024 Apr 1;183(6):2539–47. doi: 10.1007/s00431-024-05508-4 (PMC11098929; doi:10.1007/s00431-024-05508-4)
Supplement: Supplementary file 2 — Supplementary file2 (DOCX 15 KB) [file 431_2024_5508_MOESM2_ESM.docx]

# **Additional search strategies:**

Orientating search strategy for breathing at birth:

("Infant, Newborn"[Mesh] OR "Newborn"[Tw] OR "Newborns"[Tw] OR "Neonate"[Tw] OR "Neonates"[Tw]) AND ("Respiratory Center"[Mesh] OR "Respiratory"[Tw] OR "Respiration"[Mesh] OR "Respiration"[Tw] OR "Respirations"[Tw] OR "Respirating"[Tw] OR "Breathing"[Tw] OR "Apnea"[Mesh] OR "Apnea"[Tw] OR "Apneas"[Tw] OR "Apnoea"[Tw] OR "Apnoeas"[Tw] OR "ventilatory"[Tw] OR "Ventilation"[Tw] OR "Ventilations"[Tw] OR "Intubation"[Mesh] OR "Intubation"[Tw] OR "Intubations"[Tw] OR "Ventilation"[Tw] OR "Ventilations"[Tw] OR "Caffeine"[Mesh] OR "Caffeine"[Tw] OR "Pulmonary Surfactants"[Mesh] OR "Surfactant"[Tw] OR "Surfactants"[Tw] OR "Oxygen Saturation"[Mesh] OR "Saturation"[Tw] OR "Saturations"[Tw] OR "Heart Rate"[Mesh] OR "Heart Rate"[Tw] OR "Heart Rates"[Tw] OR "Hypoxia"[Mesh] OR "Hypoxia"[Tw] OR "Hypoxic"[Tw] OR "Oxygen"[Mesh] OR "Oxygen"[Tw] OR "oxygenation"[tw] OR "oxygenations"[tw] OR "Hypoxemia"[Tw] OR "Hypoxemias"[Tw] OR "Hypoxaemia"[Tw] OR "Hypoxaemias"[Tw] OR "Hypoxemic"[Tw] OR "Hypoxaemic"[Tw] OR "Anoxia"[Tw] OR "Anoxemia"[Tw] OR "Anoxaemia"[Tw] OR "Resuscitation"[Mesh] OR "Resuscitation"[Tw] OR "Resuscitations"[Tw] OR "CPR"[Tw] OR "bag and mask"[Tw] OR "chest compression"[Tw] OR "chest compressions"[Tw] OR "Epinephrine"[Mesh] OR "Epinephrine"[Tw] OR "adrenaline"[Tw] OR "depression"[Tw] OR "depressions"[Tw] OR "depressed"[Tw] OR "Continuous Positive Airway Pressure"[Mesh] OR "Continuous Positive Airway Pressure"[Tw] OR "CPAP"[Tw]) AND "transition"[Tw] AND "birth"[Tw]

706 records 21/12/2023

Acetaminophen and breathing neonate:

("Acetaminophen"[Mesh] OR "Acetaminophen"[Tw] OR "Hydroxyacetanilide"[Tw] OR "APAP"[Tw] OR "p-Acetamidophenol"[Tw] OR "p-Hydroxyacetanilide"[Tw] OR "Paracetamol"[Tw] OR "N-(4-Hydroxyphenyl)acetanilide"[Tw] OR "Acetamidophenol"[Tw] OR "N-Acetyl-p-aminophenol"[Tw] OR "Acephen"[Tw] OR "Acetaco"[Tw] OR "Tylenol"[Tw] OR "Anacin-3"[Tw] OR "Anacin 3"[Tw] OR "Datril"[Tw] OR "Panadol"[Tw] OR "Acamol"[Tw] OR "Algotropyl"[Tw]) AND ("Infant, Newborn"[Mesh] OR "Newborn"[Tw] OR "Newborns"[Tw] OR "Neonate"[Tw] OR "Neonates"[Tw] OR "Fetus"[Mesh] OR "Fetus"[Tw] OR "Foetus"[Tw] OR "Fetuses"[Tw] OR "Foetuses"[Tw] OR "Fetal"[Tw] OR "Foetal"[Tw]) AND ("Respiratory Center"[Mesh] OR "Respiratory"[Tw] OR "Respiration"[Mesh] OR "Respiration"[Tw] OR "Respirations"[Tw] OR "Respirating"[Tw] OR "Breathing"[Tw] OR "Apnea"[Mesh] OR "Apnea"[Tw] OR "Apneas"[Tw] OR "Apnoea"[Tw] OR "Apnoeas"[Tw])

123 records 21/12/2023

Acetaminophen and chorioamnionitis:

("Acetaminophen"[Mesh] OR "Acetaminophen"[Tw] OR "Hydroxyacetanilide"[Tw] OR "APAP"[Tw] OR "p-Acetamidophenol"[Tw] OR "p-Hydroxyacetanilide"[Tw] OR "Paracetamol"[Tw] OR "N-(4-Hydroxyphenyl)acetanilide"[Tw] OR "Acetamidophenol"[Tw] OR "N-Acetyl-p-aminophenol"[Tw] OR "Acephen"[Tw] OR "Acetaco"[Tw] OR "Tylenol"[Tw] OR "Anacin-3"[Tw] OR "Anacin 3"[Tw] OR "Datril"[Tw] OR "Panadol"[Tw] OR "Acamol"[Tw] OR "Algotropyl"[Tw]) AND ("Chorioamnionitis"[Mesh] OR "Chorioamnionitis"[Tw] OR "amnionitis"[Tw] OR "funisitis"[Tw] OR "intrauterine infection"[Tw] OR "intrauterine infections"[Tw] OR "intrauterine inflammations"[Tw] OR "intrauterine inflammation"[Tw] OR "intra uterine infection"[Tw] OR "intra uterine infections"[Tw] OR "intra uterine inflammations"[Tw] OR "intra uterine inflammation"[Tw] OR "Triple I"[Tw] OR "intraamniotic infection"[Tw] OR "intraamniotic infections"[Tw] OR "intra-amniotic infection"[Tw] OR "intra-amniotic infections"[Tw] OR "Prenatal inflammation"[Tw] OR "Perinatal inflammation"[Tw] OR "antenatal inflammation"[Tw] OR "Prenatal infection"[Tw] OR "Perinatal infection"[Tw] OR "antenatal infection"[Tw] OR "Prenatal infections"[Tw] OR "Perinatal infections"[Tw] OR "antenatal infections"[Tw] OR "placental inflammation"[Tw] OR "placental inflammations"[Tw] OR "placental inflammatory lesion"[Tw] OR "placental inflammatory lesions"[Tw] OR "placental infection"[Tw] OR "placental infections"[Tw])

123 records 21/12/2023

N-Acetylcysteine and breathing neonate:

("Acetylcysteine"[Mesh] OR "Acetylcysteine"[Tw] OR "Acetyl cysteine"[Tw] OR "Acetyl L cysteine"[Tw]) AND ("Infant, Newborn"[Mesh] OR "Newborn"[Tw] OR "Newborns"[Tw] OR "Neonate"[Tw] OR "Neonates"[Tw] OR "Fetus"[Mesh] OR "Fetus"[Tw] OR "Foetus"[Tw] OR "Fetuses"[Tw] OR "Foetuses"[Tw] OR "Fetal"[Tw] OR "Foetal"[Tw]) AND ("Respiratory Center"[Mesh] OR "Respiratory"[Tw] OR "Respiration"[Mesh] OR "Respiration"[Tw] OR "Respirations"[Tw] OR "Respirating"[Tw] OR "Breathing"[Tw] OR "Apnea"[Mesh] OR "Apnea"[Tw] OR "Apneas"[Tw] OR "Apnoea"[Tw] OR "Apnoeas"[Tw])

382 records 21/12/2023

N-Acetylcysteine and chorioamnionitis:

("Acetylcysteine"[Mesh] OR "Acetylcysteine"[Tw] OR "Acetyl cysteine"[Tw] OR "Acetyl L cysteine"[Tw]) AND ("Chorioamnionitis"[Mesh] OR "Chorioamnionitis"[Tw] OR "amnionitis"[Tw] OR "funisitis"[Tw] OR "intrauterine infection"[Tw] OR "intrauterine infections"[Tw] OR "intrauterine inflammations"[Tw] OR "intrauterine inflammation"[Tw] OR "intra uterine infection"[Tw] OR "intra uterine infections"[Tw] OR "intra uterine inflammations"[Tw] OR "intra uterine inflammation"[Tw] OR "Triple I"[Tw] OR "intraamniotic infection"[Tw] OR "intraamniotic infections"[Tw] OR "intra-amniotic infection"[Tw] OR "intra-amniotic infections"[Tw] OR "Prenatal inflammation"[Tw] OR "Perinatal inflammation"[Tw] OR "antenatal inflammation"[Tw] OR "Prenatal infection"[Tw] OR "Perinatal infection"[Tw] OR "antenatal infection"[Tw] OR "Prenatal infections"[Tw] OR "Perinatal infections"[Tw] OR "antenatal infections"[Tw] OR "placental inflammation"[Tw] OR "placental inflammations"[Tw] OR "placental inflammatory lesion"[Tw] OR "placental inflammatory lesions"[Tw] OR "placental infection"[Tw] OR "placental infections"[Tw])

28 records 21/12/2023

Cord clamping and chorioamnionitis:

("Umbilical Cord Clamping"[Mesh] OR "cord clamping"[Tw] OR "cord clamp"[Tw] OR "placental transfusion"[Tw] OR "intact cord"[Tw]) AND ("Chorioamnionitis"[Mesh] OR "Chorioamnionitis"[Tw] OR "amnionitis"[Tw] OR "funisitis"[Tw] OR "intrauterine infection"[Tw] OR "intrauterine infections"[Tw] OR "intrauterine inflammations"[Tw] OR "intrauterine inflammation"[Tw] OR "intra uterine infection"[Tw] OR "intra uterine infections"[Tw] OR "intra uterine inflammations"[Tw] OR "intra uterine inflammation"[Tw] OR "Triple I"[Tw] OR "intraamniotic infection"[Tw] OR "intraamniotic infections"[Tw] OR "intra-amniotic infection"[Tw] OR "intra-amniotic infections"[Tw] OR "Prenatal inflammation"[Tw] OR "Perinatal inflammation"[Tw] OR "antenatal inflammation"[Tw] OR "Prenatal infection"[Tw] OR "Perinatal infection"[Tw] OR "antenatal infection"[Tw] OR "Prenatal infections"[Tw] OR "Perinatal infections"[Tw] OR "antenatal infections"[Tw] OR "placental inflammation"[Tw] OR "placental inflammations"[Tw] OR "placental inflammatory lesion"[Tw] OR "placental inflammatory lesions"[Tw] OR "placental infection"[Tw] OR "placental infections"[Tw])

25 records 21/12/2023

Anti-inflammatory agents and chorioamnionitis:

("Anti-Inflammatory Agents"[Mesh] OR "Anti-Inflammatory"[Tw] OR "Anti Inflammatory"[Tw] OR "Anti Inflammatories"[Tw] OR "Antiinflammatories"[Tw]) AND ("Chorioamnionitis"[Mesh] OR "Chorioamnionitis"[Tw] OR "amnionitis"[Tw] OR "funisitis"[Tw] OR "intrauterine infection"[Tw] OR "intrauterine infections"[Tw] OR "intrauterine inflammations"[Tw] OR "intrauterine inflammation"[Tw] OR "intra uterine infection"[Tw] OR "intra uterine infections"[Tw] OR "intra uterine inflammations"[Tw] OR "intra uterine inflammation"[Tw] OR "Triple I"[Tw] OR "intraamniotic infection"[Tw] OR "intraamniotic infections"[Tw] OR "intra-amniotic infection"[Tw] OR "intra-amniotic infections"[Tw] OR "Prenatal inflammation"[Tw] OR "Perinatal inflammation"[Tw] OR "antenatal inflammation"[Tw] OR "Prenatal infection"[Tw] OR "Perinatal infection"[Tw] OR "antenatal infection"[Tw] OR "Prenatal infections"[Tw] OR "Perinatal infections"[Tw] OR "antenatal infections"[Tw] OR "placental inflammation"[Tw] OR "placental inflammations"[Tw] OR "placental inflammatory lesion"[Tw] OR "placental inflammatory lesions"[Tw] OR "placental infection"[Tw] OR "placental infections"[Tw])

322 records 22/02/2024
